# Outcomes After Repeat Alcohol Septal Ablation in Patients With Obstructive Hypertrophic Cardiomyopathy

**DOI:** 10.1016/j.jacadv.2026.102595

**Published:** 2026-02-14

**Authors:** Takashi Hiruma, Mitsunobu Kitamura, Itaru Takamisawa, Ryo Abe, Tosei Kawai, Takashi Funaki, Yuki Izumi, Ryosuke Higuchi, Junya Matsuda, Yukichi Tokita, Mamoru Nanasato, Nobuo Iguchi, Tomohiro Iwakura, Tomoki Shimokawa, Shuichiro Takanashi, Hiroo Takayama, Yuichi J. Shimada, Hitoshi Takano, Kuniya Asai, Mitsuaki Isobe, Morimasa Takayama

**Affiliations:** a Department of Cardiology, Sakakibara Heart Institute, Tokyo, Japan; b Hypertrophic Cardiomyopathy Center, Sakakibara Heart Institute, Tokyo, Japan; c Department of Cardiovascular Medicine, Graduate School of Medicine, The University of Tokyo, Tokyo, Japan; d Department of Cardiovascular Medicine, Nippon Medical School, Tokyo, Japan; e Department of Cardiovascular Surgery, Sakakibara Heart Institute, Tokyo, Japan; f Department of Cardiovascular Surgery, Kawasaki Saiwai Hospital, Tokyo, Japan; g Division of Cardiac, Vascular & Thoracic Surgery, Columbia University Medical Center, New York, New York, USA; h Division of Cardiology, Department of Medicine, Columbia University Medical Center, New York, New York, USA; i Sakakibara Heart Institute, Tokyo, Japan

**Keywords:** alcohol septal ablation, hypertrophic cardiomyopathy, left ventricular obstruction, repeat procedure, septal reduction therapy

## Abstract

**Background:**

Alcohol septal ablation (ASA) may necessitate a repeat procedure if the obstructive myocardium is not sufficiently ablated; however, the outcomes after repeat ASA are not well studied.

**Objectives:**

The objective of the study was to evaluate outcomes in patients with obstructive hypertrophic cardiomyopathy after repeat ASA.

**Methods:**

Of the 663 patients with obstructive hypertrophic cardiomyopathy who underwent ASA, 84 with repeat ASA were analyzed. Residual left ventricular (LV) obstruction was stratified according to proximal or distal obstruction. The primary outcome was the absence of symptomatic residual LV obstruction, defined as NYHA functional class I or LV gradient <50 mm Hg, or both at 12 months after repeat ASA. Factors associated with the primary outcome were assessed using logistic regression. Secondary outcomes included cardiovascular mortality, fatal arrhythmia, heart failure hospitalization, and 3rd intervention.

**Results:**

Of the 84 patients, 41 had proximal and 43 had distal obstruction. Five patients failed to achieve technical success. One patient with proximal obstruction died due to infection during the index hospitalization. At 12 months after repeat ASA, 80.7% (67/83) achieved the primary endpoint: 90.0% (36/40) with proximal obstruction and 72.1% (31/43) with distal obstruction. The primary outcome was associated with proximal obstruction (adjusted OR: 4.06; 95% CI: 1.09-18.3). During a median follow-up of 7.1 years, there were 13 deaths (15.5%). Cardiovascular mortality and heart failure hospitalization were similar between those with proximal or distal obstruction, whereas fatal arrhythmia and 3rd interventions were more frequent in distal obstruction.

**Conclusions:**

The site of residual obstruction may be important for understanding long-term outcomes after repeat ASA, but this requires further studies.

In patients with obstructive hypertrophic cardiomyopathy (oHCM), the primary treatment goal is to relieve the left ventricular (LV) outflow tract obstruction.^1^^,^^2^ Recently, myosin inhibitors have emerged as alternatives to septal reduction therapy (SRT) for managing symptoms related to LV obstruction.^3^ Surgical myectomy has been established as the gold standard of SRT for decades.^4^, ^5^, ^6^ On the other hand, myocardial ablation by ethanol injection has limitations owing to the septal branch (SB) territories. The relation between the candidate SB and extent of obstructive septal hypertrophy is crucial for achieving optimal results with alcohol septal ablation (ASA). However, identifying the myocardial territory perfused by the candidate branch remains challenging, particularly when the vessel is a small sub-branch.^7^ A considerable number of patients who undergo ASA experience symptomatic residual LV obstruction and often require repeat SRT. The reported reintervention rate after ASA ranges from 7.1% to 18.2%, which is notably higher than that after myectomy, where the rate is generally <2%.^7^, ^8^, ^9^, ^10^, ^11^, ^12^, ^13^, ^14^, ^15^, ^16^, ^17^, ^18^

The therapeutic approach for repeat SRT needs to be individualized, integrating the spatial relation between the surviving SB and culprit myocardium causing residual LV obstruction. Indeed, some patients present with proximal obstruction, whereas others exhibit distal obstruction after regression of the ablated myocardium. Both anatomical phenotypes can be caused by geometric uncoverage of the culprit obstructive myocardium or progression of septal hypertrophy. For these anatomical types, repeat ASA may mitigate LV obstruction owing to uncovered myocardium. However, the association between the mechanism of residual LV obstruction and outcomes of repeat ASA has not been fully investigated. Therefore, this study aimed to evaluate the impact of the anatomical patterns of residual LV obstruction on procedural and clinical outcomes after repeat ASA.

## Methods

### Study design and population

We analyzed the clinical and procedural data of patients from a two-center registry who underwent ASA for the treatment of oHCM. This registry included 663 consecutive patients (415 from Sakakibara Heart Institute, Tokyo, and 248 from Nippon Medical School, Tokyo, Japan) who underwent ASA between January 1998 and November 2022. Among 101 patients who underwent repeat SRT after the initial ASA, 84 who underwent repeat ASA were included in the analysis. The candidates were prospectively registered after the initial ASA procedure. Clinical information was retrospectively collected from hospital medical records of the 2 participating centers and evaluated by cardiologists specializing in the HCM treatment. The patient characteristics included demographic information, symptoms, presence of arrhythmia and/or cardiac implantable devices, comorbidities, prescribed medications, history of hospitalization for heart failure, previous cardiac surgery, previous catheter-based intervention, echocardiographic parameters, cardiac magnetic resonance imaging measurements, procedural details of ASA, and follow-up data. Follow-up evaluations were routinely scheduled before discharge, and at 1, 6, and 12 months, and annually thereafter to obtain the same clinical information and echocardiographic parameters as those collected at baseline (Supplemental Appendix).

This study was approved by the institutional ethics committee (approval number: 21-055), and informed consent was obtained from all participants in accordance with the principles of the Declaration of Helsinki.

### Multimodal evaluation for residual LV obstruction

Residual LV obstruction after ASA was comprehensively assessed using echocardiography and contrast-enhanced cardiac magnetic resonance imaging, both of which were performed at each center. In addition, invasive hemodynamics were reevaluated for an accurate assessment of the gradient and LV function.

#### Echocardiography

The mechanisms of residual LV obstruction, intra-LV gradient, systolic anterior motion (SAM) of the mitral leaflet and mitral regurgitation were assessed using Doppler echocardiography. The Valsalva maneuver was executed as the provocation method. In patients with a gradient >100 mm Hg at rest, provocation testing was not performed. SAM was graded as follows: none, mild (leaflet-septal distance >10 mm), moderate (leaflet-septal distance ≤10 mm, or brief mitral leaflet-septal contact <30% of the systolic duration), and severe (leaflet-septal contact ≥30% of the systolic duration) according to the American Society of Echocardiography guidelines.^19^ Structural anomalies of the LV and mitral valve complex, as well as intrinsic valvular heart diseases, were thoroughly evaluated.^20^^,^^21^ In patients with an undetermined diagnosis of structural anomalies or concomitant valvular heart disease, transesophageal echocardiography was performed as appropriate.

#### Cardiac magnetic resonance

Cine imaging was routinely performed to evaluate the extent of hypertrophy and identify structural anomalies in the LV, except in patients with claustrophobia or implantable cardiac devices not compatible with magnetic resonance imaging. The residual culprit myocardium which was not ablated at the initial ASA was evaluated using cine and delayed enhancement imaging, except in patients with allergic reactions to contrast agents and severe chronic kidney disease.

#### Invasive hemodynamic evaluation

Intra-LV pressure was measured using a specially designed 4-F or 5-F catheter with a small pigtail shape and distally positioned side holes (mTAKA, Nipro Corp). Another diagnostic catheter was placed in the ascending aorta to measure the aortic pressure. To evaluate the entire intra-LV gradient, the pigtail catheter was positioned at the LV apex, and the peak aortic pressure was measured at rest, during the Valsalva maneuver, and postextrasystole. If a relevant gradient (≥50 mm Hg) was observed, pressure recordings were sequentially obtained at the LV inflow, apex, mid-cavity, and subaortic levels by repositioning the pigtail catheter to identify the site of the residual obstruction.

In patients with chronic atrial fibrillation, the intra-LV gradient was calculated by averaging 5 consecutive beats to minimize beat-to-beat variability.

### Definitions of the mechanisms of residual LV obstruction after ASA

Based on the imaging evaluation for the level of LV obstruction, the mechanisms were categorized as follows.

**Figure 1:**
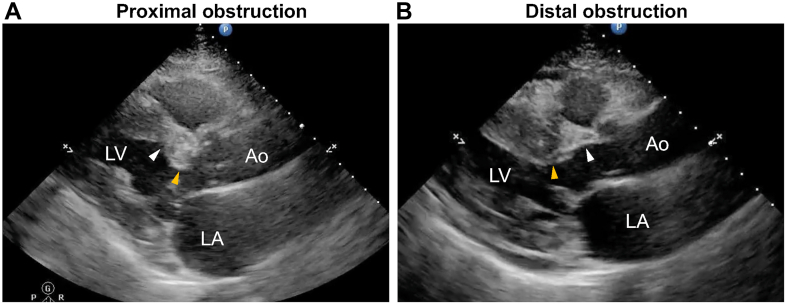
**Mechanisms of Residual Obstruction at the Left Ventricular Outflow Tract** (A) Representative echocardiographic image of proximal obstruction. The previously ablated myocardium (white arrowhead) shows regression with no LV obstruction, whereas systolic anterior motion of the mitral leaflet occurs at the level of the uncovered myocardium (orange arrowhead) proximal to the previous ablation site, resulting in residual proximal obstruction. (B) Representative echocardiographic image of distal obstruction. The previously ablated myocardium (white arrowhead) shows regression with no LV obstruction, whereas systolic anterior motion of the mitral leaflet occurs at the level of the uncovered myocardium (orange arrowhead) distal to the previous ablation site, resulting in residual distal obstruction. Ao = Aorta; LA = left atrium; LV = left ventricle.
2) *Distal obstruction*: residual basal-to-mid LV obstruction at the mitral leaflet coaptation level distal to the previous ablation site (Figure 1B, Video 2).

1) *Proximal obstruction*: residual subaortic LV obstruction at the outflow level proximal to the previous ablation site and no distal LV obstruction (Figure 1A, Video 1).

### Identification of candidate SBs for repeat ASA

Candidate SBs for ethanol injection were determined using angiography. Principally, the first major SB was ablated during the first procedure. When the first major SB completely disappeared, the other SBs and unusual branches were the main targets of the repeat procedure. Given the residual sub-branches of the initially ablated SBs, the same branch was treated to fully permeate ethanol into each sub-branch.

Myocardial territories perfused by the target SBs were confirmed using myocardial contrast echocardiography performed during branch occlusion. The balloon size for branch occlusion was determined by its diameter; if the diameter was <1 mm, a microcatheter was carefully used for target branch selection. Following angiographic evaluation, interventional cardiologists with expertise in ASA discussed the suitability of each candidate SB for ethanol injection.

### Therapeutic decision-making for repeat SRT

Before evaluation for repeat SRT, medical treatment was optimized according to the current guidelines;^4^^,^^5^ however, cardiac myosin inhibitors were not available during the study period. Repeat SRT was indicated for patients who met the same criteria as those used for the initial ASA: specifically, an intra-LV gradient ≥50 mm Hg at rest or under provocation, as measured by Doppler echocardiography, in combination with NYHA functional class II to IV or recurrent syncopal episodes. Patients with LV systolic dysfunction (ejection fraction <50%) were excluded from SRT. Treatment selection for repeat SRT was determined by the institutional heart team comprising cardiologists, cardiac surgeons, radiologists, and anesthetists. No pacemaker implantation was conducted for the purpose of reducing the intra-LV gradient.

Repeat ASA was considered feasible in patients with residual LV obstruction caused by residual proximal or distal obstruction in relation to the previous ablation site, after excluding patients with intracardiac structures unfeasible for ASA. These structures included muscular bundles inserted into the basal septum, anterior displacement of the papillary muscle, and direct insertion of the papillary muscle into the anterior mitral leaflet.^20^, ^21^, ^22^, ^23^ Intrinsic mitral regurgitation was surgically treated with mitral valve repair or replacement when improvement in SAM-related mitral regurgitation after septal regression was unlikely. Patients without suitable SBs for ethanol injection were considered eligible for myectomy.

### Repeat ASA procedure

Repeat ASA procedures were performed by experienced operators (I.T., M.K., H.T., and Y.T.) who were proctored by a common senior operator (M.T.) to standardize the protocol at the 2 participating institutions. The repeat procedures conformed to de novo ASA protocol, as described elsewhere (Supplemental Appendix).^8^^,^^14^ In brief, all procedures were guided by contrast-enhanced intraprocedural transthoracic echocardiography to confirm that the ethanol was delivered exclusively to the culprit septum. Pure ethanol was injected into the target SBs at a speed of 0.3 mL/min until complete ablation of the culprit septum was confirmed on echocardiography. Once optimal septal ablation was achieved, the disappearance of the target SBs without ethanol misplacement or vessel injury was confirmed using coronary angiography. Finally, the intra-LV gradient was evaluated at rest, during the Valsalva maneuver, and postextrasystole.

Technical success was defined as homogeneous ethanol deposition within the culprit septum without leakage into the unplanned myocardial region. Procedural success was defined as technical success with a peak-to-peak intra-LV gradient <30 mm Hg by catheter at the end of the procedure and the absence of in-hospital death.

### Imaging protocol after repeat ASA

All patients underwent standard evaluations at discharge, and at 1, 6, and 12 months after repeat ASA, followed by annual checkups. Each follow-up included medical interviews, blood tests, and electrocardiography. NYHA functional class was recorded at 1, 6, and 12 months after repeat ASA. Transthoracic echocardiography was performed at discharge, and at 6 and 12 months after repeat ASA. For annual follow-up, evaluations were accepted within a ±3-month window around each scheduled time point to accommodate differences in visit timing.

### Endpoints

The primary endpoint was the absence of symptomatic residual LV obstruction at 12 months after repeat ASA: defined as achievement of an intra-LV gradient <50 mm Hg at rest or with provocation, or NYHA functional class I with no recurrent syncopal episodes irrespective of the gradient. The primary endpoint was evaluated in 83 patients who were eligible for assessment at 12 months after repeat ASA, after excluding 1 patient who died during the index hospitalization.

Long-term secondary outcomes included changes in NYHA functional class, cardiovascular death, fatal arrhythmic events, hospitalization for heart failure, and 3rd SRT. NYHA functional class II was subdivided into class IIs (slight limitation of physical activity) and class IIm (moderate limitation) to provide a more granular assessment of symptom severity.^24^ Cardiovascular death was defined as death related to fatal arrhythmic events, heart failure, stroke, or procedural complications. Fatal arrhythmic events were defined as a composite of sudden cardiac death, ventricular fibrillation, sustained ventricular tachycardia, and appropriate discharge of an implantable cardioverter-defibrillator. Follow-up data were collected from electronic medical records or by telephone when necessary.

### Statistical analysis

Continuous variables are expressed as median (IQR) and were compared using the Mann-Whitney *U* test. Categorical variables are presented as numbers with percentages and were compared using the chi-square test or Fisher exact test, as appropriate. Paired continuous variables were compared using the Wilcoxon paired test, and paired categorical variables were compared using the McNemar test. Multivariable logistic regression analysis was performed to elucidate the impact of residual proximal LV obstruction on the primary endpoint, with adjustment for age, maximal LV wall thickness, intra-LV gradient, and mitral regurgitation as potential risk factors for residual LV obstruction.^7^^,^^15^ Cardiac events were plotted using cumulative incidence curves with 95% CIs. Comparisons were performed using the Gray test for cardiovascular death when noncardiovascular death was considered a competing risk, and for fatal arrhythmic events, hospitalization for heart failure, and 3rd SRT when all-cause death was considered a competing risk. Statistical significance was defined as a 2-sided *P* value <0.05. All statistical analyses were conducted using R software (R Foundation for Statistical Computing).

## Results

### Study population

**Figure 2:**
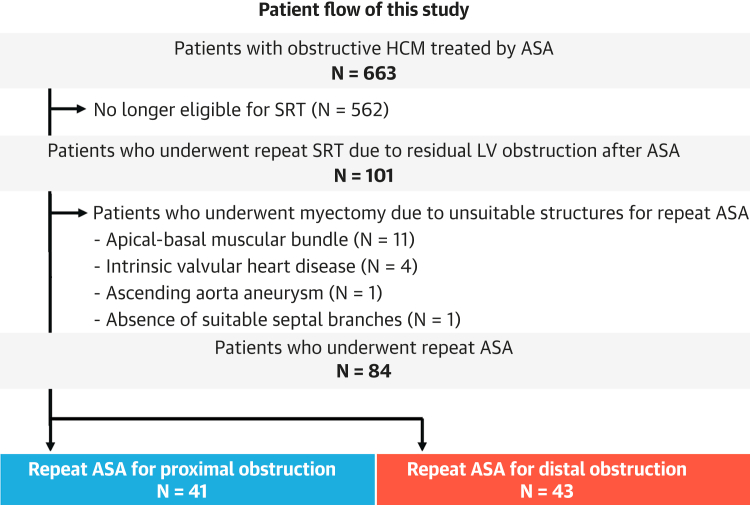
**Patient Flow of This Study** Among the 663 patients with obstructive HCM treated with ASA, 101 underwent repeat SRT for residual LV obstruction. Seventeen patients with structures unsuitable for repeat ASA underwent myectomy. Of the 84 patients who underwent repeat ASA, 41 had proximal obstruction and the remaining 43 had distal obstruction. ASA = alcohol septal ablation; HCM = hypertrophic cardiomyopathy; SRT = septal reduction therapy; other abbreviation as in Figure 1.

Among 663 patients previously treated with ASA, 101 (15.2%) underwent repeat SRT (Figure 2). Of these 101 patients, 17 (16.8%) were treated with surgical myectomy, and the remaining 84 (83.2%) underwent repeat ASA. In the evaluation of the mechanisms, proximal obstructions were identified in 41 of the 84 patients who underwent repeat ASA (48.8%), whereas distal obstruction was observed in the remaining 43 patients (51.2%). The median period from the initial ASA to repeat ASA was 1.6 (IQR: 0.9-3.2) years.

The demographic data stratified by mechanism are shown in Table 1. Patients with distal obstruction had greater maximal LV wall thickness, a higher prevalence of mid-cavity obstruction, a lower SAM grade, and a lower prevalence of procedural success at the initial ASA than those of patients with proximal obstruction. Other demographic data were comparable between the groups.

**Table 1:** Baseline Demographics of Study Cohort at Repeat ASA

	All (N = 84)	Proximal Obstruction (n = 41)	Distal Obstruction (n = 43)	*P* Value
Demographic characteristics				
Age, y	67 (54-75)	68 (58-76)	64 (43-71)	0.085
Female, %	59 (70.2)	31 (75.6)	28 (65.1)	0.34
Body mass index, kg/m^2^	24 (21-26)	24 (21-27)	24 (21-26)	0.94
Family history of HCM/sudden cardiac death, %	22 (26.2)	8 (19.5)	14 (32.6)	0.22
NYHA functional class				-
I, %	1 (1.2)	1 (2.4)	0 (0.0)	
IIs, %^a^	39 (46.4)	21 (51.2)	18 (41.9)	
IIm, %^a^	29 (34.5)	13 (31.7)	16 (37.2)	
III, %	12 (14.3)	4 (9.8)	8 (18.6)	
IV, %	3 (3.6)	2 (4.9)	1 (2.3)	
Atrial fibrillation				0.66
Paroxysmal, %	14 (16.7)	8 (19.5)	6 (14.0)	
Persistent, %	2 (2.4)	1 (2.4)	1 (2.3)	
Chronic, %	1 (1.2)	1 (2.4)	0 (0.0)	
Prior pacemaker, %	4 (4.8)	0 (0.0)	4 (9.3)	-
Prior implantable cardioverter, %	6 (7.1)	3 (7.3)	3 (7.0)	1.00
Hypertension, %	41 (48.8)	19 (46.3)	22 (51.2)	0.67
Diabetes, %	5 (6.0)	4 (9.8)	1 (2.3)	0.20
Dyslipidemia, %	44 (52.4)	22 (53.7)	22 (51.2)	0.83
Chronic kidney disease, %	10 (11.9)	6 (14.6)	4 (9.3)	0.52
Medications				
Beta-blocker, %	81 (96.4)	39 (95.1)	42 (97.7)	0.61
Calcium-channel blocker, %	15 (17.9)	8 (19.5)	7 (16.3)	0.78
Class Ia antiarrhythmic agents, %	71 (84.5)	35 (85.4)	36 (83.7)	1.00
Class III antiarrhythmic agents, %	7 (8.3)	4 (9.8)	3 (7.0)	0.71
Echocardiographic parameters				
Interventricular septal wall thickness, mm	15 (13-17)	15 (12-16)	15 (13-20)	0.035
LV posterior wall thickness, mm	12 (10-13)	11 (10-13)	12 (10-13)	0.35
Maximum LV wall thickness, mm	17 (15-20)	16 (14-17)	19 (17-21)	<0.001
LV end-diastolic diameter, mm	42 (37-48)	43 (39-48)	41 (35-47)	0.33
LV end-systolic diameter, mm	26 (22-30)	26 (25-31)	25 (21-30)	0.22
LV ejection fraction, %	65 (62-69)	65 (61-67)	65 (62-70)	0.58
Left atrial diameter, mm	44 (38-48)	45 (39-50)	42 (38-47)	0.080
Pressure gradient at rest, mm Hg	80 (54-108)	80 (59-109)	74 (39-108)	0.49
LV obstruction type				<0.001
Outflow tract obstruction, %	52 (61.9)	37 (90.2)	15 (34.9)	
Midventricular obstruction, %	7 (8.3)	0 (0.0)	7 (16.3)	
Combined obstruction, %	25 (29.8)	4 (9.8)	21 (48.8)	
Systolic anterior motion of mitral leaflet				0.031
None, %	8 (9.5)	0 (0.0)	8 (18.6)	
Mild, %	15 (17.9)	7 (17.1)	8 (18.6)	
Moderate, %	59 (70.2)	33 (80.5)	26 (60.5)	
Severe, %	2 (2.4)	1 (2.4)	1 (2.3)	
Mitral regurgitation				-
Grade 0, %	15 (17.9)	4 (9.8)	11 (25.6)	
Grade 1+, %	29 (34.5)	13 (31.7)	16 (37.2)	
Grade 2+, %	28 (33.3)	17 (41.5)	11 (25.6)	
Grade 3+, %	12 (14.3)	7 (17.1)	5 (11.6)	
Grade 4+, %	0 (0.0)	0 (0.0)	0 (0.0)	
Procedural data of the initial ASA				
Number of the septal branches ablated				0.11
Single septal branch, %	48 (58.5)	27 (67.5)	21 (50.0)	
Multiple septal branches, %	34 (41.5)	13 (32.5)	21 (50.0)	
Volume of the injected ethanol, ml^b^	2.3 (1.7-3.0)	2.0 (1.7-2.8)	2.6 (1.7-4.0)	0.23
Postprocedural peak creatine kinase, U/L^b^	951 (747-1,299)	921 (781-1,162)	996 (696-1,388)	0.40
Intra-LV gradient measured by catheter				
Pregradient at rest, mm Hg	86 (34-121)	80 (32-119)	94 (50-122)	0.27
Postgradient at rest, mm Hg	24 (8-46)	19 (8-33)	31 (6-64)	0.12
Pregradient on Valsalva maneuver, mm Hg	100 (69-124)	97 (67-132)	103 (72-119)	0.97
Postgradient on Valsalva maneuver, mm Hg	47 (19-86)	47 (14-67)	41 (22-94)	0.50
Technical success, %	77 (91.7)	38 (92.7)	39 (90.7)	1.00
Procedural success, %	48 (57.1)	29 (70.7)	19 (44.2)	0.017

Values are median (IQR) or n (%).

*P* values indicate comparisons between patients with proximal and distal obstruction.

ASA = alcohol septal ablation; HCM = hypertrophic cardiomyopathy; LV = left ventricular.

a Patients with NYHA functional class II were stratified into 2 groups: those with slight limitation (IIs) and moderate limitation (IIm) in physical activity.

b 2 patients (1 for proximal obstruction and 1 for distal obstruction) were excluded because none of the septal branches were ablated during the initial ASA due to wiring failure.

### Repeat ASA procedures

The procedural details of the repeat ASA are listed in Table 2. The target branches of the 2 mechanisms differed significantly. The most-proximal SB and same SB as the previously ablated SB were more frequently ablated for proximal obstruction, whereas the distal SB was more frequently ablated for distal obstruction. Detailed data on the targeted branches at repeat ASA are shown in Supplemental Table 1. The postprocedural LV gradient at rest was low in patients with proximal obstruction. A representative case of the same (sub-branch) ablation for residual proximal obstruction is shown in Supplemental Figure 2.

**Table 2:** Details and Procedural Outcomes of Repeat ASA

	All (N = 84)	Proximal Obstruction (n = 41)	Distal Obstruction (n = 43)	*P* Value
Period from the initial ASA, y	1.9 (1.0-3.5)	2.0 (1.0-4.1)	1.4 (0.8-2.5)	0.14
Number of the septal branch ablated	1 (1-2)	1 (1-2)	1 (1-2)	0.10
Multiple septal branch ablation, %	31 (36.9)	12 (29.3)	19 (44.2)	0.18
The most proximal septal branch ablation, %	36 (42.9)	23 (56.1)	13 (30.2)	0.027
The same septal branch ablation, %	29 (34.5)	19 (46.3)	10 (23.3)	0.038
Distal septal branch ablation, %	43 (51.2)	12 (29.3)	31 (72.1)	<0.001
Origin of the ablated septal branch				
Left anterior descending artery, %	67 (79.8)	29 (70.7)	38 (88.4)	0.044
Nonleft anterior descending artery, %	22 (26.2)	13 (31.7)	9 (20.9)	0.26
Volume of the injected ethanol, ml^a^	2.1 (1.5-2.9)	2.0 (1.5-2.7)	2.3 (1.5-3.3)	0.20
Post procedural peak creatine kinase, U/L^a^	782 (456-1,026)	735 (453-1,021)	824 (457-1,116)	0.64
Intra-LV gradient measured by catheter				
Pregradient at rest, mm Hg	54 (20-85)	40 (19-87)	63 (24-85)	0.31
Postgradient at rest, mm Hg	16 (6-41)	11 (4-31)	19 (12-48)	0.016
Pregradient on Valsalva maneuver, mm Hg	80 (36-111)	80 (57-110)	79 (34-120)	0.74
Postgradient on Valsalva maneuver, mm Hg	38 (11-64)	34 (6-55)	40 (14-85)	0.17
Minor complications				
Right bundle branch block, %	27 (32.1)	14 (34.2)	13 (30.2)	0.82
Left bundle branch block, %	0 (0.0)	0 (0.0)	0 (0.0)	-
Left axis deviation, %	5 (6.0)	3 (7.3)	2 (4.7)	0.67
Transient advanced or complete AVB, %	13 (15.5)	8 (19.5)	5 (11.6)	0.38
Major complications				
Permanent pacemaker implantation, %	3 (3.6)	2 (4.9)	1 (2.3)	0.61
Cardiac tamponade, %	0 (0.0)	0 (0.0)	0 (0.0)	-
Coronary artery dissection/perforation, %	3 (3.6)	2 (4.9)	1 (2.3)	0.61
Ethanol leakage to the unplanned myocardium, %	0 (0.0)	0 (0.0)	0 (0.0)	-
Sustained ventricular tachyarrhythmias, %	0 (0.0)	0 (0.0)	0 (0.0)	-
Cardiogenic shock requiring mechanical support, %	0 (0.0)	0 (0.0)	0 (0.0)	-
Access site complication, %	0 (0.0)	0 (0.0)	0 (0.0)	-
Failed wiring for targeted septal branch, %	2 (2.4)	1 (2.4)	1 (2.3)	1.00
Technical success, %	79 (94.0)	38 (92.7)	41 (95.4)	0.61
Procedural success, %	56 (66.7)	31 (75.6)	25 (58.1)	0.090
In-hospital death, %	1 (1.2)	1 (2.4)	0 (0.0)	-

Values are median (IQR) or n (%).

*P* values indicate comparisons between patients with proximal and distal obstruction.

AVB = atrioventricular block; other abbreviations as in Table 1.

a 2 patients who underwent repeat ASA for proximal obstruction were excluded because none of the septal branches were ablated during repeat ASA.

An advanced or complete atrioventricular block developed in 8 of the 41 patients with proximal obstruction (19.5%; 95% CI: 8.8%-34.9%) and in 5 of the 43 with distal obstruction (11.6%; 95% CI: 3.9%-25.1%), of whom 2 of the 41 with proximal obstruction (4.9%; 95% CI: 0.6%-16.5%) and 1 of the 43 with distal obstruction (2.3%; 95% CI: 0.0%-12.2%) necessitated permanent pacemaker implantation, respectively.

Five of the 84 patients who underwent repeat ASA (6.0%; 95% CI: 2.0%-13.3%) failed to achieve technical success. Reasons for procedural failure of repeat ASA included SB dissection in 2 patients, guidewire perforation of SB into the myocardium in 1, and failure to cross the guidewire in 2, as detailed in Supplemental Table 2. Of these 5 patients, 2 were converted to myectomy, 1 underwent ASA via alternative SB arising from the diagonal branch, and 2 were medically treated without additional SRT. No other adverse events related to procedure were observed. Although the technical success rates were comparable, the procedural success rate was numerically higher in patients with proximal obstruction than in those with distal obstruction. One of the 41 patients with proximal obstruction (2.4%; 95% CI: 0.0%-12.9%) died of septic shock on postprocedural day 7, whereas no in-hospital deaths occurred among patients with distal obstruction.

### Improvements of hemodynamic status and symptoms

**Figure 3:**
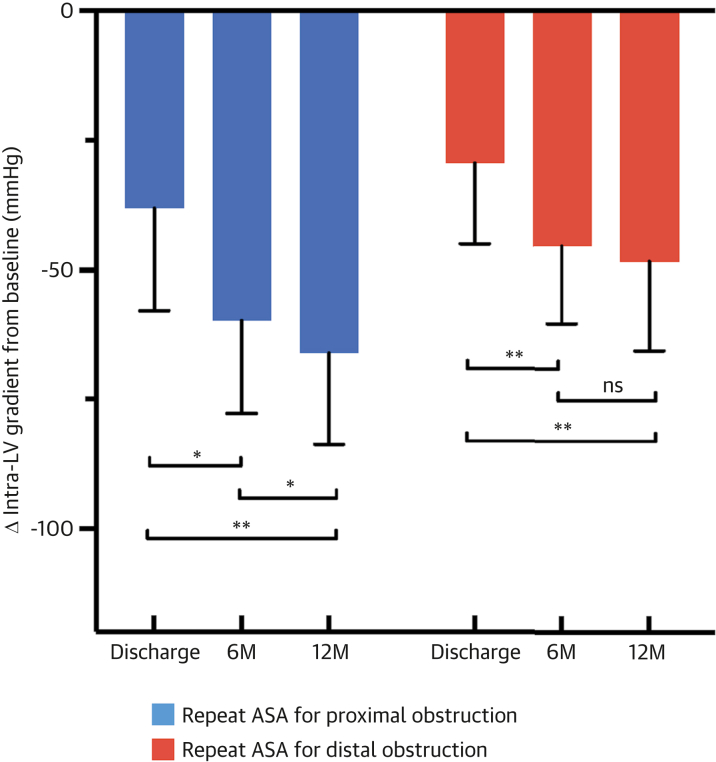
**Changes in Intra-Left Ventricle Gradient** Bars represent changes in the intra-LV gradient at discharge and at 6 and 12 months after repeat ASA. Patients with proximal obstruction (blue bars) showed a gradual reduction in the intra-LV gradient from discharge to the 12-month follow-up, whereas those with distal obstruction (red bars) experienced a reduction from discharge to the 6-month follow-up but did not show further improvement by the 12-month time point. ∗*P* < 0.05; ∗∗*P* < 0.01; ns = not statistically significant; error bars represent 95% CIs. Abbreviations as in Figures 1 and 2.

**Figure 4:**
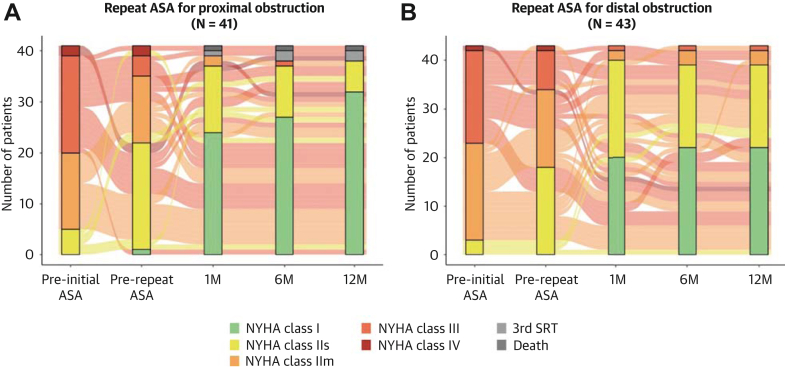
**Changes in NYHA Functional Class** Symptom status according to NYHA functional class is shown as alluvial plot at preinitial ASA, pre-repeat ASA, and at 1-month, 6-month, and 12-month after repeat ASA (A) in patients with proximal obstruction, and (B) in those with distal obstruction. Patients with NYHA functional class II were stratified into 2 groups: those with slight limitation (IIs) and moderate limitation (IIm) in physical activity. Abbreviations as in Figure 2.

The intra-LV gradient continuously reduced beyond 6 months after repeat ASA for proximal obstruction, whereas no further improvement was observed after repeat ASA for distal obstruction (Figure 3). At 12 months, the intra-LV gradients were different; 17 mm Hg (IQR: 14-40 mm Hg) after repeat ASA for proximal obstruction and 34 mm Hg (IQR: 17-54 mm Hg) after repeat ASA for distal obstruction (*P* = 0.034). NYHA functional class improved by at least 1 grade in 83.3% (95% CI: 69.8%-92.5%) after repeat ASA for proximal obstruction and 79.1% (95% CI: 64.0%-90.0%) after repeat ASA for distal obstruction (Figure 4). Nevertheless, repeat ASA for distal obstruction was associated with residual heart failure symptoms with NYHA functional class II or higher at 12 months, compared with repeat ASA for proximal obstruction (*P* = 0.039). At 12 months after repeat ASA, 67 of the 83 patients (1 patient who died during index hospitalization was excluded from the primary endpoint analysis) who underwent repeat ASA (80.7%; 95% CI: 70.6%-88.6%) achieved the primary endpoint: 36 of 40 patients with proximal obstruction (90.0%; 95% CI: 76.3%-97.3%) and 31 of 43 with distal obstruction (72.1%; 95% CI: 56.6%-84.4%) (Central Illustration). Proximal obstruction was independently associated with the primary endpoint even after the adjustment for covariates (adjusted OR: 4.06; 95% CI: 1.09-18.3; *P* = 0.037) (Table 3).

**Central Illustration:**
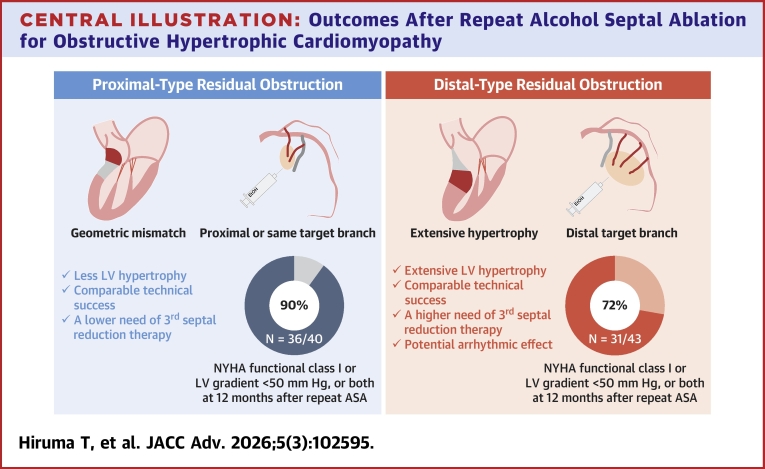
**Outcomes After Repeat Alcohol Septal Ablation for Obstructive Hypertrophic Cardiomyopathy** Residual LV obstruction was categorized as proximal or distal obstruction according to whether it involved the basal or apical portion of the initially ablated septum. Proximal obstruction occurs when basal hypertrophy persists despite the first major septal branches ablation during the initial ASA, reflecting a geometric mismatch between targeted septal branches and culprit myocardium. Distal obstruction occurs when distal hypertrophy persists despite ablation of the standard target septal branches, owing to extensive hypertrophy that cannot be fully addressed during the initial ASA. The primary endpoint, defined as achievement of NYHA functional class I or LV gradient <50 mm Hg, or both at 12 months after repeat ASA, was achieved in 90% and 72% of patients with proximal and distal obstruction. Distal obstruction is more likely to leave residual gradients and thus carries a higher likelihood of requiring additional interventions. ASA = alcohol septal ablation; LV = left ventricle.

**Table 3:** Univariate and Multivariable Analysis for the Primary Endpoint

	Univariate		Multivariable	
	OR (95% CI)	*P* Value	Adjusted OR	*P* Value
Age, per 10 y	0.90 (0.61-1.26)	0.56	0.80 (0.52-1.15)	0.24
Maximal LV wall thickness, per 1 mm increase	0.89 (0.77-1.03)	0.13	0.92 (0.77-1.11)	0.38
Intra-LV gradient, per 1 mm Hg increase	0.99 (0.98-1.00)	0.14	0.99 (0.98-1.01)	0.34
Mitral regurgitation, grade 2+ or greater	0.63 (0.20-1.89)	0.41	0.54 (0.15-1.92)	0.34
Residual proximal obstruction	3.48 (1.09-13.5)	0.035	4.06 (1.09-18.3)	0.037

The primary endpoint was defined as the absence of symptomatic residual LV obstruction at 12 months after repeat ASA: defined as achievement of an intra-LV gradient <50 mm Hg at rest or with provocation, or NYHA functional class I with no recurrent syncopal episodes irrespective of the gradient. The primary endpoint was evaluated in 83 patients who were eligible for assessment at 12 months after repeat ASA, after excluding 1 patient who died during the index hospitalization.

Abbreviation as in Table 1.

### Long-term outcomes following repeat SRT

**Figure 5:**
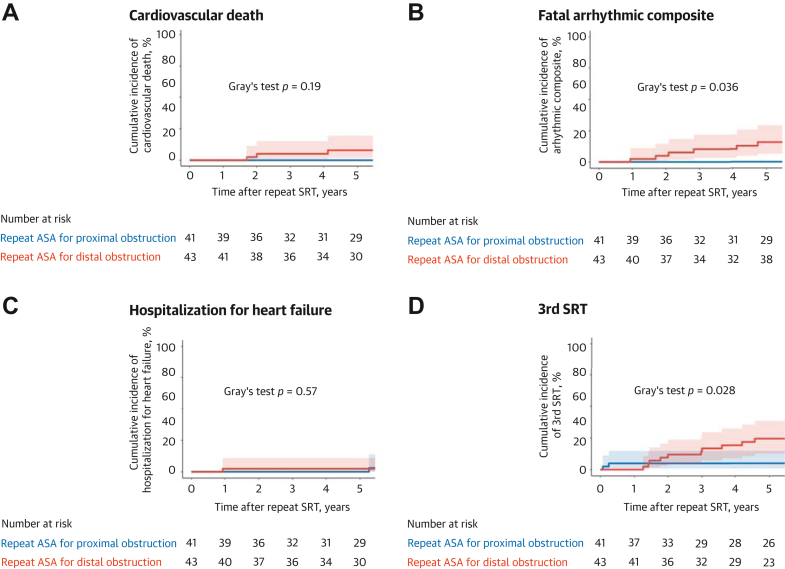
**Cumulative Incidence of Study Endpoints** Cumulative incidence plots with 95% CIs for (A) cardiovascular death, (B) fatal arrhythmic events, (C) hospitalization for heart failure, and (D) 3rd SRT. Abbreviations as in Figure 2.

During a follow-up of 7.4 (IQR: 4.1-11.7) years, there were 13 deaths (15.5%; 95% CI: 8.5%-25.0%) in the overall population of 84 patients. Six patients died from cardiovascular causes, including 5 from sudden cardiac death and 1 procedure-related death. The other 7 patients died from noncardiovascular causes. Sustained ventricular tachycardia was documented in 3 patients and ventricular fibrillation in 2 patients. Three patients were hospitalized for heart failure. The incidences of cardiovascular death and hospitalization for heart failure were comparable between the groups (Figure 5). Notably, fatal arrhythmic events were more frequent after repeat ASA for distal obstruction than after repeat ASA for proximal obstruction (Gray test *P* = 0.036). The total amount of injected ethanol and postprocedural peak creatine kinase levels throughout the initial and repeat ASA were not associated with any cardiac events (Supplemental Figures 3 and 4).

Third SRT was performed in 15 patients (17.9%; 95% CI: 10.4%-27.7%) in the overall population of 84 patients, including 2 of the 41 with proximal obstruction (4.9%; 95% CI: 6.0%-16.5%) and 13 of the 43 with distal obstruction (30.2%; 95% CI: 17.2%-46.1%) (Gray test *P* = 0.028) (Figure 5). The median period between 2nd ASA and 3rd SRT was 3.0 years (IQR: 1.4-4.6). Of these 15 patients who underwent 3rd SRT, 10 (66.7%; 95% CI: 38.4%-88.2%) underwent myectomy, where 5 exhibited obstructive muscular bundles after regression of the hypertrophied septum. A representative case is shown in Supplemental Figure 5. The other 5 patients had no suitable SBs for ASA. The remaining 5 of the 15 patients (33.3%; 95% CI: 11.8%-61.6%) had suitable SBs for additional ethanol injection and underwent 3rd ASA. There were no in-hospital deaths, and all 15 patients were completely relieved of their residual obstruction after 3rd SRT.

## Discussion

This study investigated the outcomes of repeat ASA in 84 patients with oHCM. Five patients failed to achieve technical success after repeat ASA. At 12 months after the repeat ASA, 80.7% of patients were asymptomatic with no significant LV obstruction. In this small cohort, we found that patients with proximal obstruction had better outcomes when compared to those with distal obstruction. During a follow-up of 7.4 years, 15.5% of the overall cohort died.

### Repeated ASA procedure for proximal obstruction

For proximal obstruction, repeat ASA often targets the residual proximal branches, including a small single branch into the proximal obstruction and residual sub-branches of the previously ablated branch. Such unusual branch selection could facilitate the feasibility of repeat ASA. Nevertheless, it is challenging to determine whether ablation of these accessory branches during the initial ASA is necessary to avoid residual LV obstruction. As chronic-phase improvements in hemodynamics and symptoms can be expected over time,^25^^,^^26^ it is also reasonable to defer additional branch ablation because of potential complications related to extensive myocardial ablation. In this context, accurate identification and precise surveillance of the most proximal SB are essential when residual LV obstruction is driven by proximal obstruction.

Technical challenges arise when treating the most proximal SBs, which are often small, tortuous, and may originate outside the left anterior descending artery. These anatomical features impede branch identification and hinder guidewire or balloon catheter manipulation. In selected cases, microcatheter-facilitated ASA has proven technically feasible for overcoming these limitations.^27^ Notably, this case showed that ablating even a small myocardial territory through the most-proximal SB produced meaningful hemodynamic and symptomatic improvement. These findings support the use of repeat ASA as an effective option for residual LV obstruction caused by proximal obstruction following initial ASA.

### Repeat ASA and distal septal obstruction

Repeat ASA for residual distal obstruction predominantly targeted distal SBs of the left anterior descending artery, achieved >90% technical success, and maintained >70% freedom from SRT eligibility at 12 months after initial ASA. Nevertheless, despite these procedural gains, the clinical outcomes in this subgroup remain poorer than those in patients with proximal obstruction, chiefly because of the high incidence of persistent LV obstruction. Contributing factors include a thicker interventricular septum, more extensive basal-to-mid ventricular hypertrophy, and persistent LV obstruction after repeat ASA.^28^

The anatomy itself also limits the efficacy of ASA in this subgroup: hypertrophy often expands from the base to apex, beyond the territory perfused by the standard target SBs. Even when ethanol reaches the culprit myocardial area, the ablated volume may still be too small to relieve the obstruction. Gradient and symptoms therefore persist, and a considerable number of patients eventually require 3rd SRT, most often surgical myectomy. These observations indicate that repeat ASA rarely provides reliable relief from residual LV obstruction in this subgroup. Accordingly, myectomy may offer a durable treatment for patients with extensive hypertrophy at high risk of residual distal LV obstruction.

Nevertheless, predicting residual distal obstruction that becomes evident only after relief of proximal obstruction remains challenging. A comparable pathophysiologic mechanism can occur following aortic valve replacement for severe aortic stenosis, when abrupt afterload reduction in a hypertrophied LV unmasks an intra-LV obstruction at outflow tract to mid-cavity level.^29^ In both settings, decompression of the principal LV outflow constraint—whether valvular or subvalvular—induces hemodynamic shift that may provoke secondary gradients within the LV cavity.

Recently, cardiac myosin inhibitors have altered the management paradigm for LV outflow tract obstruction. Nevertheless, their efficacy in patients with extensive hypertrophy, including those with residual obstruction after ASA, remains uncertain. Future studies should evaluate the therapeutic role of these agents in mitigating persistent gradient after ASA and define their integration with catheter-based and surgical strategies.

### Study Limitations

We acknowledge several limitations in this study. First, the study population was relatively small, which may have limited the statistical power of the findings, and the study was retrospectively designed without a validation cohort. This highlights the need for larger, multicenter, and prospective validation studies to confirm our findings. Second, all SRT procedures were performed by experienced operators at high-volume tertiary centers, which may limit the generalizability of our findings when applied to low-volume centers. Third, genetic testing was not performed in this analysis. Given the genotype-phenotype correlations, incorporating genetic data may help explain why patients with distal obstruction exhibit worse clinical outcomes and optimize patient selection for repeat SRT. Fourth, echocardiographic information on the mitral valve complex was limited to mitral regurgitation severity and SAM. Integrating detailed imaging assessments of other mitral valve abnormalities may further improve patient selection for repeat SRT. Finally, emerging cardiac myosin inhibitors have changed clinical decision-making and may reduce the need for SRT. Future studies should focus on defining an optimal strategy for managing residual LV obstruction.

## Conclusions

Repeat ASA is often effective for patients with residual proximal obstruction and suitable SB and no other obstructive structures. However, late risks, including mortality, are significant. Distal obstruction at the time of repeat ASA may be associated with worse outcomes, but further larger studies are needed to truly understand the impact of site and mechanisms of LV obstruction and outcomes.

Perspectives **COMPETENCY IN MEDICAL KNOWLEDGE AND PATIENT CARE:** Although repeat ASA can relieve residual LV obstruction, its success may vary according to the underlying site of obstruction.

**TRANSLATIONAL OUTLOOK:** In the era of cardiac myosin inhibitors, the role of ASA requires redefinition. Future studies should aim to identify patients unresponsive to myosin inhibition and refine SRT indications and strategies.

## Funding support and author disclosures

This study was supported by Sakakibara Heart Foundation Cardiovascular Research Program 2023, Clinical Research Project for Hypertrophic Cardiomyopathy and the part-time researcher fund of the Sakakibara Heart Foundation. The authors have reported that they have no relationships relevant to the contents of this paper to disclose.

## Supplementary material

Video 1 Representative Doppler echocardiographic image of proximal obstruction. The previously ablated myocardium shows akinesis and regression. Doppler flow acceleration begins at the level of the uncovered myocardium proximal to the previous ablation site.

Video 2 Representative Doppler echocardiographic image of distal obstruction. The previously ablated myocardium shows akinesis and regression. Doppler flow acceleration begins at the level of the uncovered myocardium distal to the previous ablation site.
