# Supplementary material for: Outcomes After Repeat Alcohol Septal Ablation in Patients With Obstructive Hypertrophic Cardiomyopathy
Source: JACC Adv. 2026 Feb 14;5(3):102595. doi: 10.1016/j.jacadv.2026.102595 (PMC12925452; doi:10.1016/j.jacadv.2026.102595)
Supplement: Supplemental_Material [file mmc3.pdf]

SUPPLEMENTAL MATERIALS

Outcomes After Repeat Alcohol Septal Ablation in Patients with Obstructive Hypertrophic Cardiomyopathy

1. Supplemental Methods

Supplemental Method 1. Collected baseline data in a multicenter HCM registry...........page 1
Supplemental Method 2. ASA procedures...page 2

2. Supplemental Tables

Supplemental Table 1. Target branches at repeat ASA in relationship to the initially ablated branches...page 4
Page 5 Supplemental Table 2. Detailed data of the patients without technical success at repeat ASA...page 5

3. Supplemental Figures

Supplemental Figure 1. Cumulative incidence of repeat ASA from the initial ASA.......page 6
Supplemental Figure 2. Representative case undergoing sub-branch ablation at repeat ASA...page 7
Page 8 Supplemental Figure 3. Hazard of all-cause mortality according to the total volume of injected ethanol volume and post-procedural peak CK levels throughout the initial and repeat ASA...page 8
Page 9 Supplemental Figure 4. Hazard of lethal arrhythmic events according to the total volume of injected ethanol volume and post-procedural peak CK levels throughout the initial and repeat ASA...page 9
Page 10 Supplemental Figure 5. Representative case undergoing surgical resection of the apical-basal muscle bundle during myectomy as 3rd SRT...page 10

1. Supplemental Methods

Supplemental Method 1. Collected baseline data in a multicenter HCM registry

Baseline characteristics included demographic information (age, sex, body mass index), family history of HCM or sudden cardiac death, New York Heart Association (NYHA) functional class, documented atrial fibrillation, presence of cardiac implantable electronic devices (permanent pacemaker and implantable cardioverter-defibrillator), comorbidities (hypertension, diabetes mellitus, dyslipidemia, coronary artery disease, and chronic kidney disease), and prescribed medications (beta-blockers [bisoprolol, carvedilol or metoprolol], non-dihydropyridine calcium-channel blockers [diltiazem or verapamil], class Ia antiarrhythmic agents [cibenzoline or disopyramide], and class III antiarrhythmic agents [amiodarone or sotalol]). History of hospitalization for heart failure, prior cardiac surgery, and previous catheter-based interventions were also recorded. A family history of HCM was determined when more than 1 family member presented with clinical evidence of HCM. A family history of sudden cardiac death included any unexpected death of a family member regardless of age.

A standardized imaging protocol was used at both centers. Transthoracic echocardiography was performed at baseline, before discharge, at 6 months and 12 months after the index ASA, and annually thereafter. Analyses included M-mode–derived measurements (interventricular septal thickness, posterior wall thickness, left ventricular (LV) end-diastolic and end-systolic diameters), LV mass index, left atrial diameter and volume index, LV ejection fraction, and intra-LV gradients at rest and with Valsalva's maneuver. Mitral regurgitation severity and systolic anterior motion of the mitral leaflet were also assessed. Echocardiographic data collected at follow-up included the same set of parameters.

Cardiac magnetic resonance imaging was performed before ASA, unless contraindicated (e.g., claustrophobia or non–MRI-compatible cardiac devices). Cine sequences were used to assess LV volumes and function. Late gadolinium enhancement imaging was used to quantify myocardial fibrosis. The same acquisition protocol was used in both institutes.

Supplemental Method 2. ASA procedures

We performed alcohol septal ablation (ASA) procedures with guidance of echocardiography to identify the appropriate ethanol injection to the culprit myocardium. An over-the-wire coronary balloon with a diameter of 1.25 to 2.5mm was utilized to inject ethanol to the target septal branch (SB). The speed of ethanol injection was 0.1 ml per 20 seconds (0.3 mL/min). If the target SB was very small with a diameter of less than 1 mm, we utilized a microcatheter (Caravel, Asahi Intec, Aichi, Japan) instead of an over-the-wire balloon. With the target small SB wedged and occluded by the microcatheter, an ethanol injection was performed without leakage of contrast into main epicardial coronary artery. In case of complete atrioventricular block, 500 mg of hydrocortisone was intravenously administered to mitigate myocardial inflammation deteriorating the conduction. If targeted myocardial site was homogeneously dyed white with widespread backscatter, ethanol injection was terminated (approximately 1 to 2 ml per a branch). Five minutes later, the over-the-wire balloon was deflated and removed with the guidewire lumen kept aspirated manually using a 50 ml syringe. Immediately after removal of the over-the-wire balloon, coronary angiography was performed to confirm disappearance of target SB and absence of ethanol misplacement or coronary vessel injury. After hemodynamic evaluation for the intra-left ventricular (LV) gradient was repeated, hemostasis of puncture sites was done using a percutaneous closure device. Within 3 hours after the procedure, all patients were transferred to the cardiac care unit for monitoring hemodynamics and procedure-related complication. Creatine kinase (CK) value was measured every 4 hours until 24 hours after the procedure. The temporary pacemaker was routinely placed at least 72 hours after the procedure. If the advanced or complete atrioventricular block prolonged thereafter, permanent pacemaker was implanted before discharge.

Technical success was defined as homogeneous ethanol deposition within the responsible myocardium without ethanol leakage to unplanned myocardial region. Procedural success was defined as achieving technical success AND a reduction of the intra-LV gradient

measured by catheter to less than 30 mmHg at the end of the procedure AND without in-hospital death. Procedure-related complications were defined as follows; transient advanced or complete atrioventricular block, new permanent pacemaker implantation, cardiac tamponade, coronary artery dissection/perforation, ethanol leakage to the unplanned myocardium, sustained ventricular tachyarrhythmias, cardiogenic shock requiring mechanical support, access site complications and procedure-related death.

2. Supplemental Tables

Supplemental Table 1. Target branches at repeat ASA in relationship to the initially ablated branches.

Original vessel of the target branches	Relationship to the ablated branches: Proximal	Same*	Distal	Total
From the LAD artery	15 (12.5)	28 (23.0)	55 (45.0)	98 (80.3)
From the non-LAD artery	21 (17.2)	0 (0.0)	3 (2.4)	24 (20.0)
From the diagonal vessel	9 (7.4)	0 (0.0)	3 (2.4)	12 (9.8)
From the high lateral vessel	8 (6.6)	0 (0.0)	0 (0.0)	8 (6.6)
From the conus vessel	4 (3.3)	0 (0.0)	0 (0.0)	4 (3.3)
Total	36 (29.5)	28 (23.0)	58 (47.5)	122 (100)

Data are expressed as number of septal branches with percentage in parentheses.
* The same septal branches include 13 branches (10.7%) originating from the initially ablated septal branches.
ASA, alcohol septal ablation; LAD, left anterior descending artery.

Supplemental Table 2. Detailed data of the patients without technical success at repeat ASA

	Case 1	*Case 2*	*Case 3*	*Case 4*	*Case 5*
Age, years					
At the initial ASA	74	70	78	64	45
At the repeat ASA	75	71	78	64	49
Sex	Female	Female	Female	Female	Male
Mechanisms of residual LV obstruction	Proximal obstruction	Proximal obstruction	Distal obstruction	Distal obstruction	Distal obstruction
Maximum LV wall thickness, mm	15	19	18	15	17
The number of the ablated branches					
At the initial ASA	1	2	1	2	1
At the repeat ASA	0	0	1	2	3
Details of the ablated branches					
At the initial ASA	S2	S2, S4	S4	S2, S3	S1
At the repeat ASA	-	-	S5	S5, S6	S1, S2, S3
Volume of the injected ethanol, ml					
At the initial ASA	2.0	5.5	1.5	5.0	2.2
At the repeat ASA	-	-	0.8	1.6	4.5
Post-procedural peak creatin kinase, U/L					
At the initial ASA	914	1651	433	2134	585
At the repeat ASA	-	-	273	777	906
Complications					
At the initial ASA	Wiring failure (S3)	None	Main branch dissection (LAD)	None	None
At the repeat ASA	Wiring failure (conus branch)	Target SB dissection (diagonal SB)	Targeted SB perforation (S5)	Wiring failure (diagonal SB)	Target SB dissection (S4b)
Additional septal reduction therapy	Myectomy	ASA for SB from diagonal branch	Myectomy	None	None
Period from the repeat ASA, days	26	91	1533		
Maximum intra-LV gradient, mmHg					
At the initial ASA	121	95	175	88	125
At the repeat ASA	169	97	50	103	133
At the last follow-up	9	4	7	106	112
NYHA functional class					
At the initial ASA	III	IV	IIm	IIm	IIs
At the repeat ASA	III	IIs	III	IIm	IV
At the last follow-up	I	I	IIs	IIs	I
Last follow-up	Alive	Alive	Alive	Cancer death	Cancer death
Period from the repeat ASA, days	3351	2528	2241	1849	677

ASA, alcohol septal ablation; LV, left ventricle; LAD, left anterior descending artery; NYHA, New York Heart Association; SB, septal branch.

3. Supplemental Figures

Supplemental Figure 1. Cumulative incidence of repeat ASA from the initial ASA.

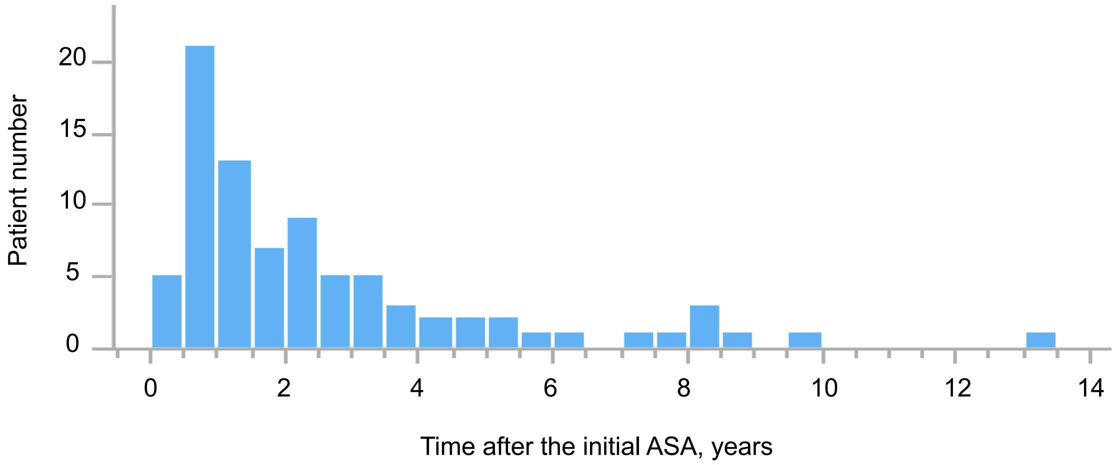

Repeat SRT was performed at 1.6 (interquartile: 0.9-3.2) years following the initial ASA
ASA, alcohol septal ablation; SRT, septal reduction therapy.

Supplemental Figure 2. Representative case undergoing sub-branch ablation at repeat ASA.

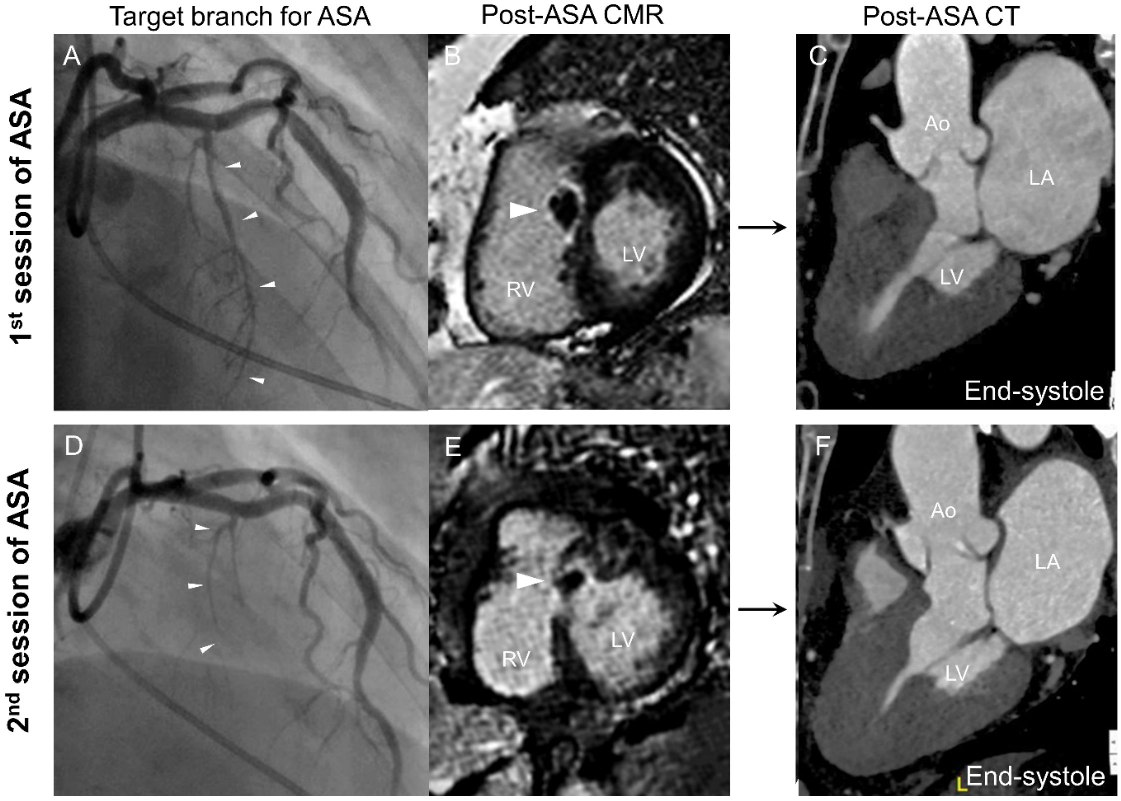

A 66-year-old female with HCM had outflow tract obstruction with an intra-LV gradient of 83 mmHg and NYHA functional class IIm. At the 1st ASA, a main branch of the first SB (arrowheads) was ablated with 2.4 ml of ethanol **(A)**. Post-procedural CK was 1068 U/L. The intra-LV gradient reduced to 32 mmHg at end of the procedure. The ablated myocardium was positioned at the right-ventricular side of the septal wall on post-ASA CMR (arrowhead) **(B)**. On post-procedural CT, the culprit myocardium insufficiently regressed with systolic anterior motion of the mitral valve leaflet remained **(C)**. After one year, the gradient increased to 72 mmHg with heart failure symptoms of NYHA functional class IIs. Residual obstruction was estimated to be caused by proximal obstruction. Therefore, the repeat ASA was performed two years and seven months after the 1st ASA. A proximal sub-branch of the first major SB was ablated with 2.8 ml of ethanol **(D)**. Post-procedural CK was elevated to 504 U/L. The gradient reduced to 23 mmHg. The ablated myocardium was positioned at the left-sided septal wall proximally adjacent to the 1st ASA on post-2nd ASA CMR **(E)**. Post-procedural CT showed marked regression of the basal LV septum **(F)**. The patient has been free from recurrence of LV obstruction and any cardiovascular events for seven years after the 2nd ASA.

Ao, aorta; ASA, alcohol septal ablation; CMR, cardiac magnetic resonance; CK, creatine kinase; CT, computed tomography; HCM, hypertrophic cardiomyopathy; LA, left atrium; LV, left ventricular; NYHA, New York Heart Association; RV, right ventricle.

Supplemental Figure 3. Hazard of all-cause mortality according to the total volume of injected ethanol volume and post-procedural peak CK levels throughout the initial and repeat ASA.

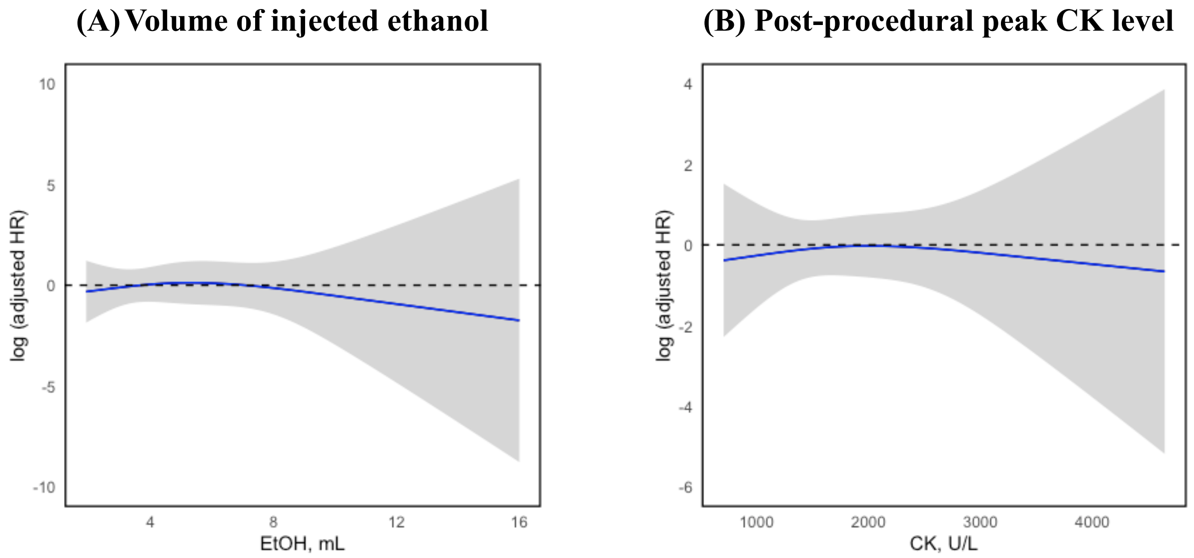

Restricted cubic splines for modeling relationships between (A) the total volume of injected ethanol and (B) post-procedural peak CK levels throughout the initial and repeat ASA on all-cause death. The model was adjusted for factors including age, sex, mechanisms of residual LV obstruction, maximal LV wall thickness, and pre-procedural intra-LV gradient. The log-transformed adjusted hazard of all-cause mortality is represented by the **blue solid line**, with the **gray shading** indicating the 95% confidence interval.

ASA, alcohol septal ablation; CK, creatine kinase; EtOH, ethanol; LV, left ventricle.

Supplemental Figure 4. Hazard of lethal arrhythmic events according to the total volume of injected ethanol volume and post-procedural peak CK levels throughout the initial and repeat ASA.

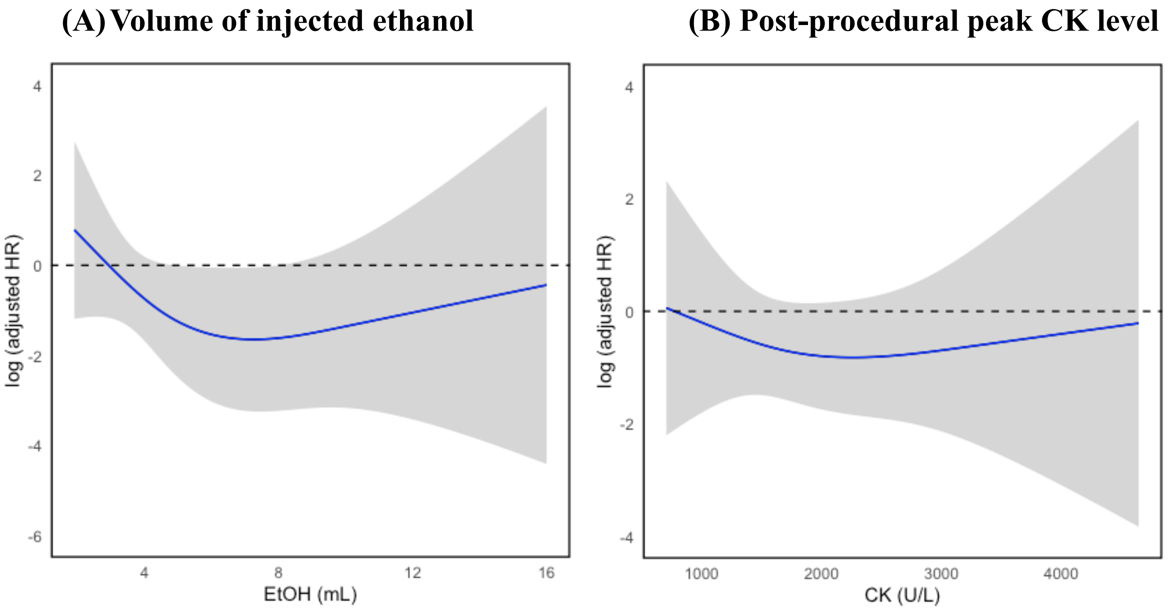

Restricted cubic splines for modeling relationships between (A) the total volume of injected ethanol and (B) post-procedural peak CK levels throughout the initial and repeat ASA on lethal arrhythmic events. The model was adjusted for factors including age, sex, mechanisms of residual LV obstruction, maximal LV wall thickness, and pre-procedural intra-LV gradient. The log-transformed adjusted hazard of lethal arrhythmic events is represented by the **blue solid line**, with the **gray shading** indicating the 95% confidence interval.
ASA, alcohol septal ablation; CK, creatine kinase; EtOH, ethanol; LV, left ventricle.

Supplemental Figure 5. Representative case undergoing surgical resection of the apical-basal muscle bundle during myectomy as 3rd SRT.

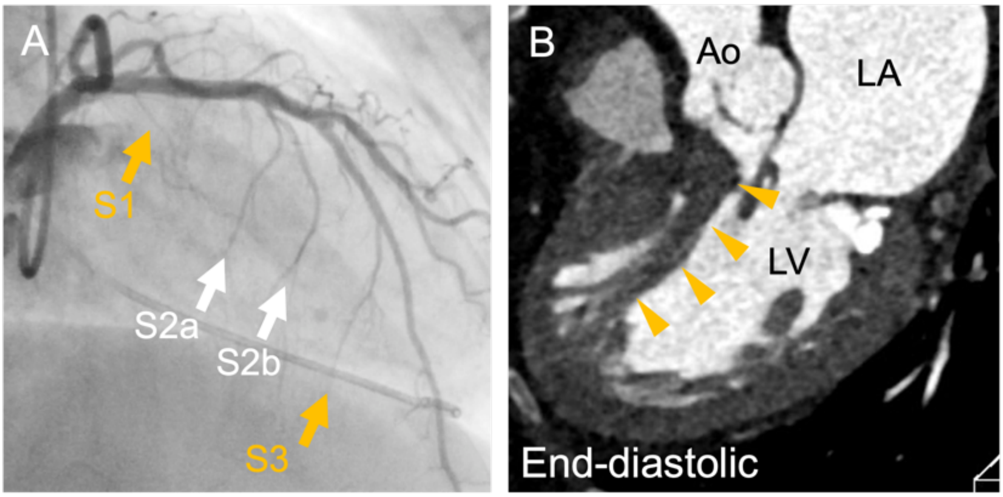

A 63-year-old female with HCM had combined obstruction at outflow tract and mid-ventricular levels. Following the 1st ASA (S1a and S2b; white arrows) and the 2nd ASA (S1 and S3; orange arrows), culprit hypertrophied septum regressed; however, thick apical-basal muscle bundles manifested (orange arrowheads). These anomalous LV structures were surgically removed combined with myectomy.
Abbreviations are same in **Supplemental Figure 2**.
